# The accuracy and feasibility of noninvasive prenatal testing in a consecutive series of 20,626 pregnancies with different clinical characteristics

**DOI:** 10.1002/jcla.24660

**Published:** 2022-09-13

**Authors:** Yunyun Zheng, Jia Li, Jianfang Zhang, Hong Yang

**Affiliations:** ^1^ Department of Obstetrics and Gynecology XiJing Hospital of Air Force Medical University Xi'an China

**Keywords:** accuracy, chromosomal microdeletion/microduplication, chromosome aneuploidies, copy number variants, feasibility, noninvasive prenatal testing, sex chromosome abnormalities

## Abstract

**Background:**

To evaluate the accuracy and feasibility of noninvasive prenatal testing (NIPT) according to the results of NIPT and pregnancy outcomes with different indications.

**Methods:**

Between October 2014 and December 2020, 20,626 pregnant women who received NIPT were included in this study. The positive predictive value (PPV) of trisomy 21, 18, and 13 (T21, T18, T13), sex chromosome abnormalities (SCAs), other chromosomal aneuploidies, and chromosomal microdeletion/microduplication were calculated. The positive results of NIPT were confirmed by amniocentesis, Karyotype analysis, and chromosome microarray analysis (CMA).

**Results:**

In total, 263 positive cases (263/20,626, 1.28%) were detected by NIPT, of which T21, T18, and T13 were 69, 26, and 9 cases, respectively. Sex chromosome abnormalities (SCAs), other chromosomal aneuploidies, and copy number variants (CNVs) were 69, 12, and 38 cases, respectively. There were true positive in 49 of T21, 13 of T18, 1 of T13, 32 of SCAs, 1 of other chromosomal aneuploidies, and 15 of CNVs. The NIPT sensitivity of T21, T18, T13, SCAs, other chromosomal aneuploidies, and CNVs was all 100%, the specialty was 99.90%, 99.94%, 99.96%, 99.82%, 99.95%, 99.89%, and the PPV was 71.01%, 50.00%, 11.11%, 46.38%, 8.33%, 39.47%, respectively. The PPV was high in T21, moderate in T18 and SCAs, and low in T13 and other chromosomal abnormalities.

**Conclusion:**

NIPT has high accuracy, specificity and and can effectively avoid the occurrence of birth defects, but it cannot replace prenatal diagnosis. The accuracy, specificity, and sensitivity of NIPT in detecting sex chromosomes, chromosome microdeletion/microduplication, and other chromosomal abnormalities should be improved.

## Graphical abstract

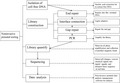

## 1 INTRODUCTION

The issue of birth defects has attracted more and more attention from scholars worldwide. Chromosomal abnormalities are main pathogenic factors for inducing birth defects.^1^ Chromosomal abnormalities include trisomy21 (T21), trisomy18 (T18), trisomy13 (T13), sex chromosome aneuploidies (SCAs), common microdeletions,^2^ rare autosomal aneuploidies, and partial deletions and duplications.^3^, ^4^, ^5^, ^6^ It is estimated that the incidences of T21, T18 and T13 are about 1/800, 1/6000 and 1/10,000, respectively.^7^, ^8^ The incidence of SCAs is about 1/500.^9^, ^10^ The incidence of chromosome abnormalities at birth is as high as 1/600.^2^ Prenatal screening is an effective intervention mean for fetal chromosomal diseases. The traditional detection methods mainly include prenatal serological screening and B‐ultrasound screening for preliminary screening and prenatal diagnosis. Due to the low detection rate and high false‐positive rate of maternal serological screening, prenatal screening technology is continuously improved and developed.^11^ Also, it is necessary to find an efficient, reliable, and economical method of prenatal pregnancy screening for use following assisted reproduction.^12^

In 2011, the massively parallel sequencing method has been used to detect maternal plasma free DNA (cfDNA). In clinical applications in China, its detection rate is as high as 99.2% for screening T21, T18, and T13 syndromes.^4^, ^13^, ^14^, ^15^ In recent years, genome‐wide screening studies have also been performed to evaluate SCAs and copy number variants (CNVs).^16^, ^17^ Clinically, noninvasive prenatal testing (NIPT) based on high‐throughput sequencing is usually used to analyze cell‐free DNA in maternal plasma to evaluate the risk for common fetal aneuploidies by quantifying the fetal chromosome complement. This valuable prenatal screening technology shows high sensitivity and specificity and is increasingly widely applied in pregnant women.^18^ In this study, we aimed to evaluate the accuracy and feasibility of NIPT according to the results of NIPT and pregnancy outcomes.

## 2 SUBJECTS AND METHODS

### 2.1 Study subjects

This study was approved by Xijing Hospital of the Air Force Medical University, China. A total of 20,626 pregnant women who voluntarily underwent NIPT from October 2014 to December 2020 were included in this study. They signed informed written consent before NIPT. Besides, they were at least 12 weeks pregnant (12–24 weeks). Information of pregnant women including age, height, weight, gestational age, pregnancy history, whether serological screening, ultrasound testing, and assisted pregnancy was recorded. Under the principle of informed consent and voluntariness, invasive prenatal diagnostic techniques were recommended for pregnant women who had poor NIPT (failed) results. Additionally, chromosomal abnormalities were analyzed. When an abnormality that matched a high‐risk NIPT diagnosis was observed through invasive prenatal diagnosis or B‐ultrasound, fetal aneuploidy with a positive NIPT result was considered. The pregnant women without abnormal results continued pregnancy. Two telephone follow‐ups were conducted to register and sort out the pregnancy outcomes.

### 2.2 cfDNA processing and sequencing

**FIGURE 1:**
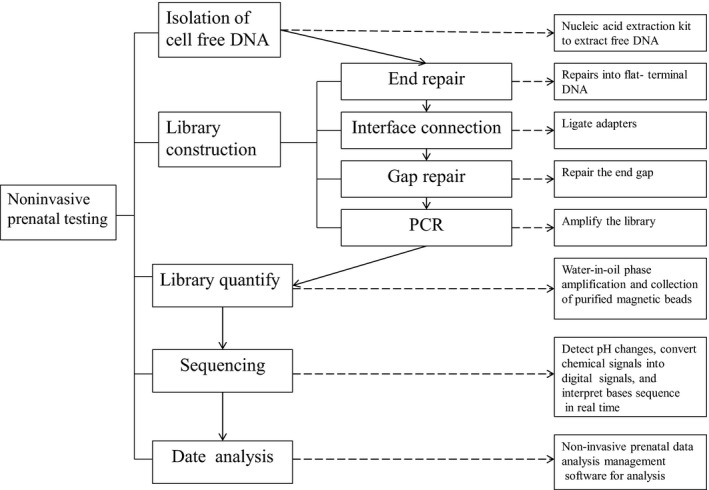
Noninvasive detection process to detect fetal free DNA in maternal plasma samples

The peripheral blood (5 ml) was obtained from pregnant women and centrifuged twice within 8 h to separate the plasma. Protocols described in previous study were strictly followed during subsequent molecular detection and bioinformatics processes, including DNA extraction, library construction, and sequencing.^5^, ^6^, ^10^ In addition to T21, T18 and T13, we presented the abnormalities of other chromosomal screenings in additional information of the subjects' reports (not shown). Figure 1 illustrated the entire testing process.

### 2.3 Karyotype analysis

All chromosomes of the sample, T21, T18, T13, sex chromosomes, and chromosome microdeletion/microduplication were analyzed. Amniocentesis was recommended for pregnant women with positive NIPT results. Karyotype analysis was performed with amniotic fluid cell culture according to the standard techniques.^19^

### 2.4 Chromosome microarray analysis (CMA)

Chromosome microarray analysis (CMA) was carried out in strict accordance with the standard.^20^ The detection process included digestion, ligation, polymerase chain reaction (PCR) amplification, fragmentation, labeling, chip hybridization, dyeing, and scanning, with AffymetrixCytogenetic Cyto Scan 750 K Array Gene Chip (Affymetrix Inc, Santa Clara, CA, USA). Databases, including DECIPHER, OMIM, DGV and UCSC, were used as references to evaluate the array data and analyze genotype–phenotype correlations.^21^

### 2.5 Follow‐up

Follow‐up of all study participants was completed from subsequent phone calls done by the Information Platform of Xijing Hospital, China. The follow‐up information included data on delivery outcomes such as survival, induced labor, or abortion, neonatological care, and the presence or absence of birth defects.

### 2.6 Statistical analysis

All statistical analyses were performed using Statistical Product and Service Solutions (SPSS) version 19.0 software (SPSS, Inc.). Data were expressed as means ± standard deviation (SD). Differences among groups were compared by analysis of variance (ANOVA). *p <* 0.05 was considered statistically significant. We used formulas to calculate the sensitivity, specificity, false‐negative rate, and predictive value as follows: sensitivity was calculated as true‐positive number/(true‐positive number + false‐negative number) × 100%; specificity was calculated as true‐negative number/(true‐negative number + false‐positive number) × 100%; the positive predictive value was calculated as true‐positive number/(true‐positive number + false‐positive number).

## 3 RESULTS

### 3.1 Clinical characteristics

There were 20,626 blood samples being sequenced. There were 36 cases (1.7%) failed to perform due to insufficient fetal scores (<4%). Blood samples of 218 (1.06%) cases were collected again because of borderline *Z*‐score or low fetal fraction, with 166 (0.80%) effective NIPT results. Finally, 20,538 subjects with the age of 27 ± 13 years and gestational age of 18 ± 5 weeks were included in this study. The age distribution and gestational age distribution are shown in Table 1.

**TABLE 1:** Characteristics of maternal age and gestational age

	Numbers	Rate (%)
Maternal age at NIPT (years)		
<20	106	0.51
20–24	1437	7.07
25–29	8041	38.98
≥30	7256	35.18
≥35	3786	18.36
Gestational age at NIPT (weeks)		
≤12	992	4.81
13–17	10,902	52.86
18–20	5810	28.17
≥21	2922	14.17

As demonstrated in Table 2, there were 263 (1.28%) pregnancies with clinically relevant chromosomal abnormalities, including 208 common aneuploidies and 55 CNVs. Before NIPT testing, pregnant women should routinely undergo screening tests, such as fetal ultrasound and maternal serum biomarker testing. Ultrasonography revealed 1468 cases (7.12%) with abnormal fetal structure, 4128 cases (20.01%) with high risk of serological screening, 719 cases (3.49%) with critical risk of serological screening, 3786 cases (18.36%) with advanced age pregnancy, 1277 cases (6.19%) without clinical indications, 360 cases (1.75%) with NT ≥3.0 mm, and 544 cases (2.64%) with trisomy 18 high‐risk. No serological screening was performed in 6695 (32.46%) cases, suggesting that 1161 pregnant women (5.63%) had a history of adverse pregnancy, 224 (1.09%) had assisted reproduction, and 264 (1.28%) had twins.

**TABLE 2:** Detection of fetal aneuploidies in different indications

Clinical features	*n* = 20,626	Rate (%)
Abnormal fetal structure	1468	7.12
High risk of serological screening	4128	20.01
Critical risk of serological screening	719	3.49
Advanced age	3786	18.36
No clinical indications	1277	6.19
NT ≥ 3.0 mm	360	1.75
Trisomy 18 high‐risk	544	2.64
No serology screening	6695	32.46
History of adverse pregnancy	1161	5.63
Assisted reproduction	224	1.09
Twins	264	1.28

*Note*: Abnormal fetal structure: malformation; High risk of serological screening: the high risk of fetal neural tube malformation or chromosomal malformation.

### 3.2 NIPT for T21, T18, T13, and SCAs

**FIGURE 2:**
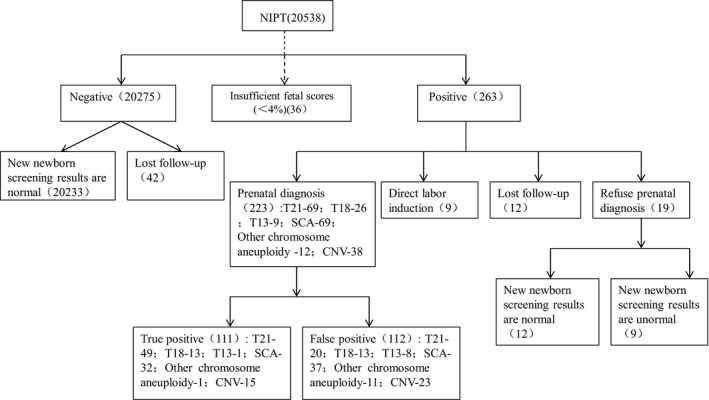
Flowchart of NIPT results and follow‐up

Of the 223 cases with prenatal diagnosis, there were 69 of T21, 26 of T18, 9 of T13, 69 of SCAs, 12 of other chromosome aneuploidy, and 38 of CNVs. A total of 49 of T21, 13 of T18, 1 of T13, 32 of SCAs, 1 of other chromosome aneuploidy, and 15 of CNV were confirmed to be true positive. The flowchart is shown in Figure 2. There were 263 abnormalities detected by NIPT, of which 223 were also detected by prenatal diagnostic testing. The cases with NIPT abnormalities were confirmed by Karyotype analysis and CMT, respectively (Table 3). There were 111 true positives and 112 false positives. The NIPT sensitivity of T21, T18, T13, SCAs, other chromosome aneuploidy, and CNVs was 100%. The specialties of them were 99.90%, 99.94%, 99.96%, 99.82%, 99.95%, 99.89%, and the PPVs of them was 71.01%, 50.00%, 11.11%, 46.38%, 8.33%, 39.47%, respectively (Table 4).

**TABLE 3:** Fetal karyotypes of NIPT positives

NIPT (*n*)	Fetal karyotypes	Numbers
T21(69)	47,XN,+21	47
	47,XN,+21/46,XN	2
	46,XN	20
T18(26)	47,XN,+18	13
	46,XN	13
T13(9)	47,XN,+13	1
	46,XN	8
SCAs(69)	45,X	6
	47,XXX	8
	47,XXY	13
	47,XYY	3
	46,X,del(X)	1
	45,X[20%]/46,XX[80%]	1
	46,XN	37

**TABLE 4:** Performance of NIPT for detecting fetal chromosomal aneuploidies

Chromosome abnormality	Positive	TP	FP	Sensitivity (%)	Specificity (%)	PPV (%)
Trisomy 21	69	49	20	100	99.90	71.01
Trisomy 18	26	13	13	100	99.94	50.00
Trisomy 13	9	1	8	100	99.96	11.11
SCAs	69	32	37	100	99.82	46.38
Other chromosome aneuploidy	12	2	10	100	99.95	8.33
CNVs	38	15	23	100	99.89	39.47

### 3.3 NIPT results for other chromosome aneuploidies and CNVs

Among the 223 pregnant women who had amniocentesis and confirmed NIPT positive results, 12 had other chromosomal abnormalities and 38 had CNV. The test results were basically consistent with the results of NIPT, and the diagnosis results were all pathogenic or possibly pathogenic. A total of 13 cases of chromosome microduplications were detected, including chromosomes 2, 3, 8, 9, 10, 13, 18, 20, and 22 (one case had both chromosome 10 duplication and chromosome 20 duplication). Among them, chromosome 22 had the highest incidence of microduplication. Three cases of chromosome microdeletion were detected, including chromosomes 10, 15, 21 (Table 5). CNVs were categorized into CNVs ≤5 Mb, 5–10 Mb, and >10 Mb groups according to the length, with 8, 3, and 6 cases, respectively.

**TABLE 5:** NIPT results for chromosome aneuploid and microdeletions/ microduplications validated by fetal Karyotyping analysis or CMA

No	Age	Weeks	NIPT indication	NIPT results	Karyotype results	CMA results
1	34	18 + 3	High risk of trisomy 21	Chr2: dup2	46,XN	Chr2:2p15 dup,2.0 Mb
2	35	14	Advanced age	Chr3: dup3	46,XN	Chr3: 3q13.31 dup,2.0 Mb
3	27	20 + 2	High risk of trisomy 21	Chr8: dup8	46,XN	Chr8:8q13.2‐ q13.3dup, 2.7 Mb
4	35	14	Advanced age	Chr8: dup8	46,XN	Chr8:3q13.31dup,2.0 Mb
5	24	18 + 1	High risk of trisomy 18	Chr9: dup9	47,XY,+9	47,XY,+9
6	32	17	No serology screening	Chr10: dup10 Chr20: dup20	46,XN	Chr10:10p15.3‐p12.1 dup, 25.5 M; Chr20:20q13.13‐q 13.33 dup, 14.2 M
7	31	16 + 1	History of adverse pregnancy Assisted reproduction	Chr13: dup13	46,XN	Chr13: 13q21dup,16.0 Mb
8	35	15 + 5	Advanced age	Chr18: dup18	46,XN	Chr18:18p11.32‐p11.21dup, 20.0 Mb
9	33	19	No serology screening	Chr22:dup22	46,XN	Chr22: 22q11.2dup,10.0 Mb
10	34	19 + 2	High risk of trisomy 21	Chr22: dup22	46,XN	Chr22:22q11.23‐q12.3dup,8.80 Mb
11	22	16 + 5	High risk of trisomy 21	Chr22: dup22	46,XN	Chr22:22q11.21dup,3.80 Mb
12	30	14	Twins	Chr22: dup22	46,XN	Chr22:22q12.3‐q13.1dup,6.60 Mb
13	28	17 + 4	High risk of trisomy 21	Chr22: dup22	46,XN	Chr22: 22q11.21del, 2.40 Mb
14	29	18 + 1	History of adverse pregnancy	Chr10: del10	46,XN	Chr10:10p14 del,3.30 Mb
15	34	19 + 2	High risk of trisomy 21	Chr15: del15	46,XN	Chr15:15q26.3del,2.90 Mb
16	34	27 + 5	Advanced age	Chr21: del21	46,XN	Chr21:21del(16‐33 Mb),18.0 Mb

## 4 DISCUSSION

At present, newborn birth defect is a worldwide problem, and there is a high incidence in China. Traditional serological prenatal screening has a 5%–50% misdiagnosis rate, and it is insensitive for other fetal chromosomal abnormalities.^9^ In recent years, chromosomal microdeletion/microduplication, sex chromosome abnormalities may be the main causes of birth defects.^22^ With the discovery of cffDNA in pregnant women, NIPT has been widely used in the screening of prenatal genetic diseases, with high diagnostic accuracy for T21, T18, and T13, and the ability to screen transgenation.^23^, ^24^, ^25^, ^26^, ^27^ In this study, the accuracy and feasibility of NIPT in 20,626 pregnant women were analyzed for providing more data support for the application of NIPT.

In this study, 263 high‐risk patients were identified by NIPT. The true‐positive rates of T21, T18, T13, SCA, other chromosome abnormalities, and CNV were 49, 13, 1, 32, 1, and 15, respectively. It was suggested that the sensitivity and specificity of NIPT for chromosome abnormalities are high. It has been reported that the PPV range of T21 is 65%–94%, T18 is 47%–85%, and T13 is 12%–62%.^28^, ^29^ Norton et al. performed NIPT on more than 15,000 pregnant women from multiple centers.^30^ Statistical analysis showed that the sensitivity and specificity of NIPT for the detection of trisomy 21 fetuses were 100%, 99%, 90%, and 100% for trisomy 18 fetuses, and 100% for trisomy 13 fetuses. Wu et al.^31^ retrospectively analyzed the NIPT test results of 11,118 pregnant women and concluded that the positive predictive values of NIPT for trisomy 21 syndrome, trisomy 18 syndrome, trisomy 13 syndrome, and sex chromosome aneuploidy were 92.16%, 91.67%, 36.36%, and 59.26%, respectively. Recently, the detection range of NIPT has been expanding and has been extended to the screening of fetal chromosome microdeletion/microduplication syndrome (MMS), namely NIPT‐plus. Our results are consistent with previous domestic studies.^30^, ^31^, ^32^ It is too early to judge the true sensitivity and false‐negative rate of SCAs in this study, because these clinical symptoms are not easily identified in neonatal examination and require long‐term prospective follow‐up.^33^ Some positive cases were proved to be false‐positive, and the causes may include low fetal score, vanishing twin, abnormal maternal chromosome,^34^, ^35^ localized placental chimera,^36^, ^37^ etc.

According to the sensitivity and specificity of NIPT to detect T21, NIPT is more accurate than serological screening. The American College of Medical Genetics and Genomics (ACMG) recommends NIPT for pregnant women to avoid the omission of more abnormalities. Pregnant women ≥35 years old are easily affected by environment, ovarian function degeneration, and egg aging. Pregnancy in the elderly is high‐risk for chromosome aneuploidy.^38^, ^39^ In our study, 3786 pregnant women ≥35 years old (18.36%) still selected NIPT, and they were high‐risk with chromosome variation during the embryonic period.

NIPT is usually carried out to screen T21, T18, T13, and sex chromosomes, and it is also gradually applied to the study of other chromosomal abnormalities and chromosome CNVs. However, there are not enough clinical data to support NIPT in chromosome deletions and duplications. More and more microdeletion and microduplication syndromes associated with phenotypes are screened, diagnosed, and studied.^34^, ^40^ In clinic, microdeletions occur more frequently than microduplications.^41^ However, there are fewer cases of microdeletions in this study. In addition, there are indeed some problems with clinical manifestations.^42^, ^43^ Therefore, we hope that the cases of this study can provide some data support.

NIPT was also used to analyze other chromosomal abnormalities and CNV screening studies. NIPT can detect microdeletion and microduplication of the fetal genome of more than 300 Kb.^44^, ^45^ In this study, 12 cases of other chromosomal abnormalities and 38 cases of CNVs were detected using NIPT technology. After prenatal diagnosis, the results of 15 cases of CNVs and one case of chromosomal abnormality were basically consistent with the results of NIPT. Among them, eight cases had CNVs ≤5 Mb, three had CNVs between 5 and 10 Mb, and six had CNVs > 10 Mb. Some subchromosomal microdeletions and microduplications have recurrent CNVs, such as 1p36, 3q, 11q23, 22q11.2 deletion syndromes.^46^, ^47^, ^48^, ^49^ In our study, there are 3q in two cases and 22q11.2 deletion syndromes in one case. After verification, all 16 cases are pathogenic or possibly pathogenic, and further follow‐up showed that the pregnancy outcomes were induced labor. It is worth noting that in our study, only 32% of deletions and duplications are related to known abnormalities. Many abnormalities may be normal genetic mutations and have no clinical significance. With the development of sequencing technology and the accumulation of clinical research, the expansion of the database may explain these unknown abnormalities. NIPT for subchromosomal microdeletion and microduplication is still in its infancy. So far, no technology has fully verified that its tests have reached a statistically significant level. Therefore, it is very important to carefully study NIPT data.

## 5 CONCLUSION

In this study, NIPT has high accuracy, specificity, and acceptance of pregnant women and can effectively avoid the occurrence of birth defects. However, it is only a prenatal screening method and cannot replace prenatal diagnosis. Therefore, it is of great significance to increase its accuracy, specificity, and sensitivity in detecting sex chromosomes, chromosome microdeletion, microduplication, and other chromosomal abnormalities in clinical practice.

## AUTHOR CONTRIBUTIONS

This work was funded by the National Key Research and Development Program of China. All the authors were involved in drafting and revising the work, obtaining, analyzing, and interpreting data. Ji Li and Hong Yang contributed to research design, clinical and data acquisition, analysis, and interpretation. All authors have provided final approval for submissions and agree to be responsible for all aspects of their work.

## Funding information

This study was supported by the National Key Research & Development Program of China (2016YFC1000700, 2016YFC1000703).

## CONFLICT OF INTERESTS

All authors declare no conflict of interests.

## CONSENT FOR PUBLICATION

None.

## Data Availability

The datasets are available from the corresponding author on reasonable request.
